# Efficacy and safety of topical nitroglycerin in the prevention of mastectomy flap necrosis: a systematic review and meta-analysis

**DOI:** 10.1038/s41598-020-63721-1

**Published:** 2020-04-21

**Authors:** Pu Wang, Luosha Gu, Zelian Qin, Qifei Wang, Jianxun Ma

**Affiliations:** 1 Department of Plastic and Reconstructive Surgery, Peking University Third Hospital, Haidian District, Beijing 100191 P. R. China; 2 Department of Plastic Surgery, The First Affiliated Hospital of Zhengzhou University, Zhengzhou, Henan 450052 P. R. China

**Keywords:** Preventive medicine, Outcomes research

## Abstract

Flap necrosis is a common complication after mastectomy, and nitroglycerin (NTG) ointment has been used successfully to treat it. However, it is not clear whether topical NTG can completely prevent the occurrence of flap necrosis after breast cancer surgery, and it is also unclear whether this treatment may cause side effects. Three randomized controlled trials (RCTs) and two retrospective cohort studies (RCSs) were included in our investigation. This meta-analysis was conducted in accordance with the Preferred Reporting Items for Systematic Reviews and Meta-Analyses (PRISMA) guidelines. We found that NTG significantly reduced the rates of mastectomy flap necrosis, full-thickness flap necrosis, and debridement as well as the rate of early complications other than flap necrosis. However, there was no significant difference in drug-related adverse reactions, explantation, superficial flap necrosis, infection, hematoma or seroma between the NTG and placebo groups.

## Introduction

Flap-related complications are extremely common after breast surgery, among them, flap necrosis is the most serious and leads to a poor prognosis. In the literature, the reported rates of mastectomy flap necrosis (MFN) range from 5% to 30%^1–9^. The rate may continue to rise as nipple-sparing mastectomy and immediate breast reconstruction (IBR) become more prevalent in young breast cancer patients, although there is some evidence that the risks of flap necrosis and implant failure are higher in IBR than in mastectomy alone^10^.

Nitroglycerin (NTG) effectively increases local blood flow by dilating arteries and veins without altering the ratio of precapillary to postcapillary resistance. Many studies have shown that NTG treatment may improve the survival of random-pattern skin flaps by increasing local blood flow^11–13^. Nonetheless, not all studies confirm the utility of NTG in skin flap preservation. Several scholars have found that daily application of NTG slow-release pads offer no greater flap survival than a control treatment^14–17^. Furthermore, some of the side effects of NTG may limit its use. However, Ricci has argued that NTG is safe and effective and does not increase the occurrence of side effects^18^.

Given the lack of consensus on the use of NTG to prevent MFN, the purpose of this meta-analysis is to determine the efficacy and safety of NTG for the prevention of MFN.

Debridement is an important solution to skin flap necrosis, but it causes psychological and physical trauma to patients. Further attention should be paid to the debridement rate after mastectomy, especially after IBR, in which circumstances it may cause the tissue expander/implant to be lost. Unfortunately, some studies have suggested that NTG cannot reduce the rate of debridement after IBR^19–21^. Hence, another purpose of this article is to determine whether NTG can reduce the flap debridement rate after mastectomy.

## Methods

Our meta-analysis was conducted in accordance with the Preferred Reporting Items for Systematic Reviews and Meta-Analyses (PRISMA) guidelines^22^. A study flow chart based on the PRISMA statement was constructed to show all literature search results.

### Search strategy and eligibility criteria

The searched databases included the following: EMBASE, PubMed and Google Scholar. The keywords consisted of “nitroglycerin” AND “breast” AND “flap necrosis”. The databases were searched from inception through May 4, 2019. Two authors (PW and LSG) independently inspected the titles and abstracts of potentially qualified studies. Any differences were discussed until a consensus was reached. In order to be included, articles needed to meet the criteria: (1) the subjects were women of any age who had a mastectomy with or without IBR; (2) NTG was used to prevent MFN; (3) the complication rates and side effects of NTG were reported. The exclusion criteria were as follows: (1) research on skin flaps at other sites, (2) comparisons between NTG and other medications to prevent flap necrosis, (3) animal experiments, and (4) review articles.

### Data extraction and quality assessment

Two reviewers extracted data from the included papers. The extracted data included the following: (1) study characteristics, (2) IBR or mastectomy alone, (3) MFN rate, (4) debridement rate, (5) loss of tissue expanders/implants, (5) NTG side effects, and (6) other early complications. The quality of potentially included studies was independently appraised by two reviewers. Studies meeting the inclusion criteria were assessed for quality by one of two methods. Randomized controlled trials (RCTs) were assessed using the Jadad scale, whereas retrospective cohort studies (RCSs) were assessed using the Newcastle-Ottawa Scale (NOS)^23,24^. RCTs scoring more than four points and RCSs scoring more than six points were regarded as high-quality studies.

### Statistical analysis

The RevMan software (the Cochrane Collaboration, Version 5.3, Oxford, UK) was used to pool the data. Dichotomous variables were presented as an OR with 95% CIs. To assess the between-study heterogeneity more precisely, the Chi^2^-based Q statistic test (Cochran’s Q statistic) was used to test for heterogeneity, and the I^2^ statistic was used to quantify the proportion of the total variation attributable to heterogeneity^23,25^. When the heterogeneity test showed I^2^ < 50%, the fixed effect model was adopted, and the random effect model was used when I^2^ ≥ 50%. Statistical analyses were performed with the software program RevMan5.3. All P-values were two-sided, and a P-value of less than 0.05 was deemed statistically significant.

### Ethical approval

This article does not contain any studies with human participants or animals performed by any of the authors.

## Results

### Literature search and study characteristics

**Figure 1:**
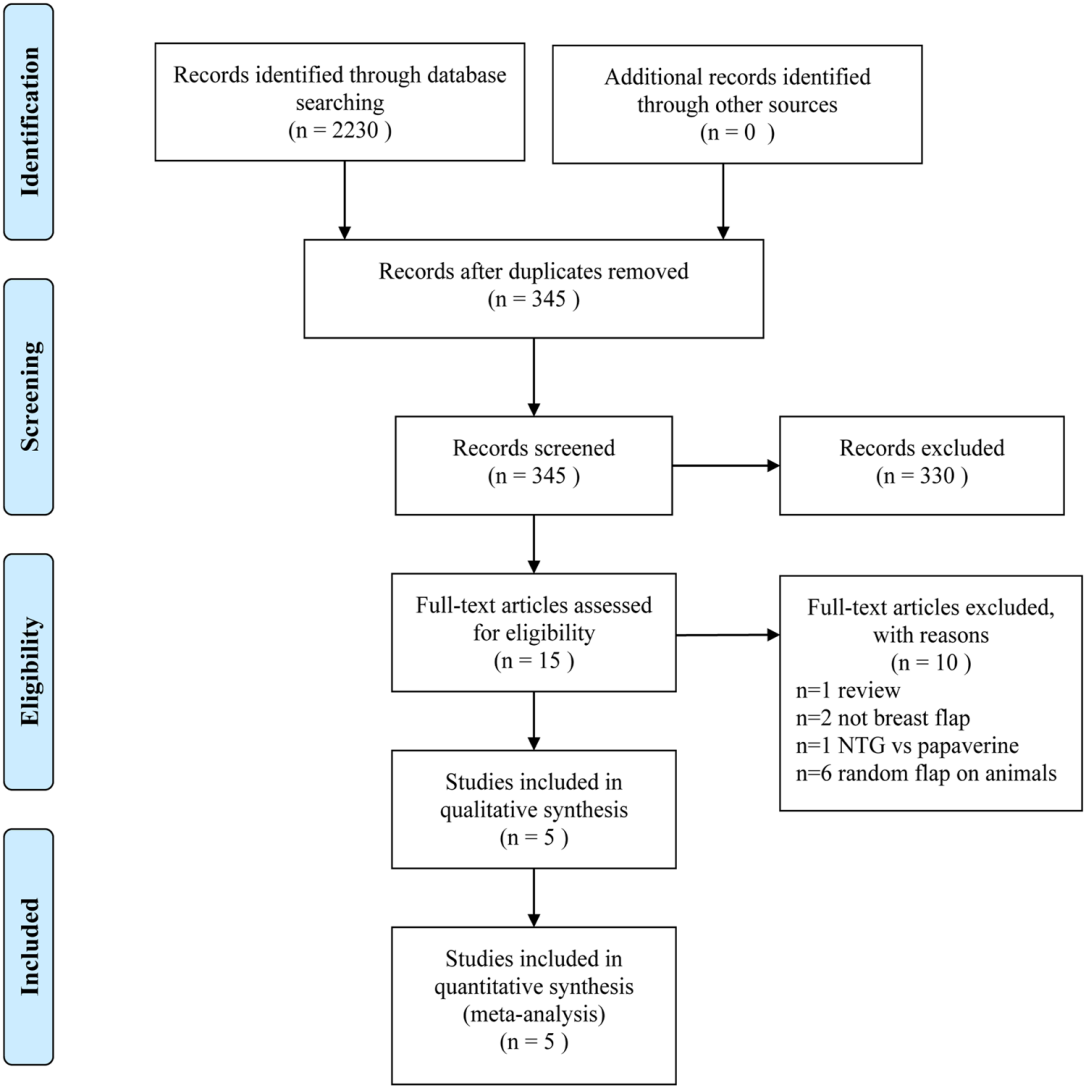
Flow diagram of study selection.

A total of 2230 potentially relevant studies were identified from the electronic databases. Based on the inclusion/exclusion criteria and quality assessment, three RCTs and two RCSs with a total of 7074 patients (IBR^19–21^: three studies with 566 patients; mastectomy alone^26,27^: two studies with 6508 patients) were ultimately included. The complete process of study selection is illustrated in Fig. 1. Summaries of the included articles (Table 1) and the quality of the included studies are shown in Tables 2 and 3.

**Table 1:** Summary of included study characteristics.

Study ID	Study Type	IBR/Mas-tectomy alone	NTG dose and types	Frequency of use	Group	NO.	Age, year	BMI, kg/m^2^	Smoking history, n(%)	Diabetes, n(%)	Hypertension, n(%)	Preoperative chemotherapy, n(%)	Preoperative radiotherapy, n(%)
Gdalevitch *et al*.^19^	RCT	IBR	45 mg, ointment	Once, keep ≥48 h	N P	85 80	50.0 ± 9.2 49.9 ± 9.7	24.9 ± 4.6 24.0 ± 4.9	18(21.2) 16(20)	0 2(2.5)	8(9.4) 11(13.8)	23 (27.1) 25 (31.2)	9 (10.6) 7 (8.8)
Fan *et al*.^26^	RCT	Mastectomy alone	5 mg, NR	NR	N P	42 40	NR NR	NR NR	NR NR	NR NR	NR NR	NR NR	NR NR
Turin *et al*.^21^	RCS	IBR	15 mg, ointment	Once, keep 3–5d	N P	158 170	48.34 ± 11.95 47.25 ± 10.92	24.6 ± 4.66 25.9 ± 5.77	18(6.5) 11(11.4)	2(1.3) 6(3.5)	10(6.3) 19(11.2)	48(30.4) 20(11.8)	2(1.3) 1(0.6)
Yun *et al*.^20^	RCS	IBR	4.5 mg, ointment	3 times (on postoperative days 2, 4,and 6)	N P	52 21	47.0 ± 9.6 45.0 ± 8.1	24.0 ± 3.7 21.0 ± 2.3	1(1.9) 2(9.5)	3 (5.8) 0	3 (5.8) 1 (4.8)	2 (3.8) 0	0 0
Kutun *et al*.^27^	RCT	Mastectomy alone	50 mg, transder-mal	5 times (5th postoperative day)	N P	3174 3252	NR NR	NR NR	NR NR	336(10.6) 413(12.7)	418(13.5) 546(16.8)	NR NR	NR NR

RCT, randomized controlled trial; RCS, retrospective cohort study; IBR, immediate breast reconstruction; NR, not reported; N, nitroglycerin; P, placebo; BMI, Body Mass Index.

**Table 2:** Quality assessment according to the Jadad scale.

References	Randomization	Concealment of allocation	Double blinding	Withdrawals and dropouts	Total
Gdalevitch *et al*.^19^	2	2	2	1	7
Fan *et al*.^26^	1	1	2	0	4
Kutun *et al*.^27^	2	1	2	0	5

**Table 3:** Quality assessment according to the NOS.

References	Selection	Comparability	Exposure	Total
Turin *et al*.^21^	3	2	3	8
Yun *et al*.^20^	3	2	3	8

### Primary outcomes

**Figure 2:**
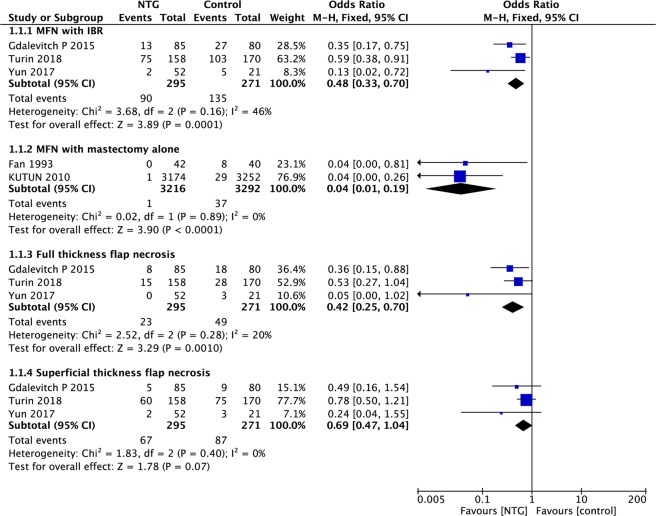
Forest plot of MFN rate.

Pooled data on MFN rates were available in all five studies. The pooled results indicated that NTG could significantly reduce the MFN rate compared with that of the control group, whether IBR was performed or not (IBR: OR, 0.48, 95% CI, 0.33–0.70, P < 0.01; I^2^ = 46%; mastectomy alone: OR, 0.04; 95% CI, 0.01–0.19; P < 0.01; I^2^ = 0%; Fig. 2).

MFN can be subdivided into full-thickness flap necrosis and superficial flap necrosis. Data for full-thickness flap necrosis and superficial flap necrosis were available in three studies of IBR. The pooled results indicated that the incidence rate of full-thickness flap necrosis was significantly lower in the NTG group than in the control group(OR, 0.42;95% CI, 0.25–0.70; P < 0.01; I^2^ = 20%; Fig. 2). However, there was no significant difference in the rate of superficial flap necrosis between the NTG and control groups (OR, 0.69; 95% CI, 0.47–1.04; P = 0.07; I^2^ = 0%; Fig. 2).

### Secondary outcomes

**Figure 3:**
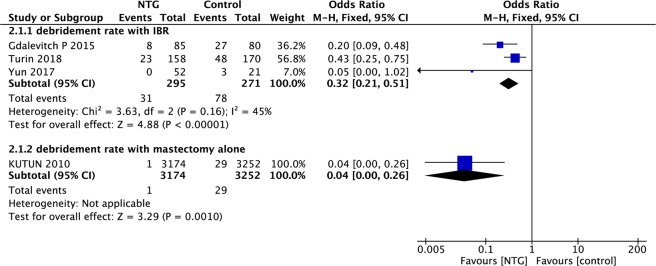
Forest plot of debridement rate.

Data on rates of debridement were extracted from 4 articles, and the incidence rate of debridement was significantly lower in the NTG group than in the control group (IBR: OR, 0.32, 95% CI, 0.21–0.51, P < 0.01; I^2^ = 45%; mastectomy alone: OR, 0.04; 95% CI, 0.00–0.26; P < 0.01; Fig. 3).

**Figure 4:**
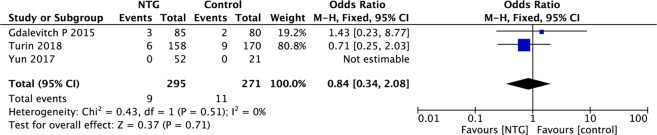
Forest plot of loss of tissue expander/implant.

In this study, data regarding explantation (loss of tissue expanders/implants) were available in three articles, and no significant intergroup difference was observed in the frequency of this event (OR, 0.84; 95% CI, 0.34–2.08; P = 0.71; I^2^ = 0%; Fig. 4).

**Figure 5:**
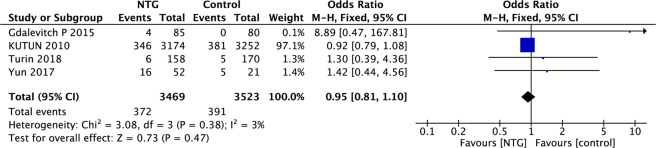
Forest plot of NTG-related adverse reactions.

Pooled data on NTG-related adverse reactions were available in four studies. The pooled results indicated that there was no significant difference in drug-related adverse reactions (such as headache, dizziness and hypotension) between NTG and the control (OR, 1.73; 95% CI, 0.79–3.77; P = 0.17; I^2^ = 3%; Fig. 5).

**Figure 6:**
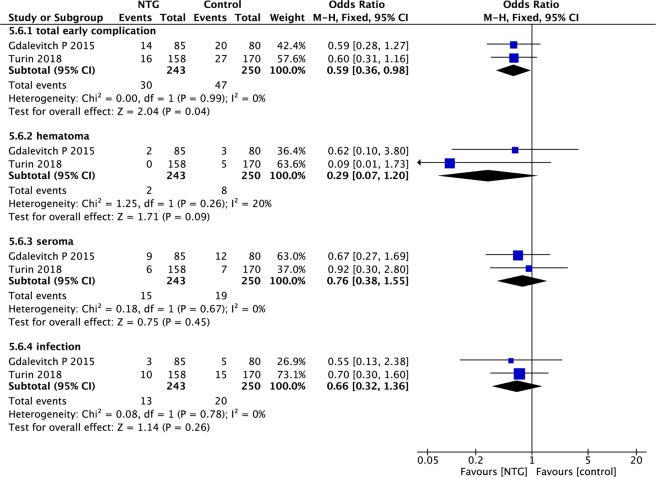
Forest plot of other early complications.

Data for other early complications were available in only two studies. The pooled results indicated that the NTG group had lower complication rates than the control group (OR, 0.59; 95% CI, 0.36–0.98; P < 0.05; I^2^ = 0%; Fig. 6).

Early complications other than flap necrosis were further subdivided into 3 categories: hematoma, seroma and infection. However, there was no significant difference in the incidence of any of those categories between the NTG and control groups (hematoma: OR, 0.29; 95% CI, 0.07–1.20; P = 0.09; I^2^ = 20%; seroma: OR, 0.76; 95% CI, 0.38–1.55; P = 0.45; I^2^ = 0%; infection: OR, 0.66; 95% CI, 0.32–1.36; P = 0.26; I^2^ = 26%; Fig. 6).

## Discussion

MFN may be affected by many factors, including smoking, age, hypertension, previous scars, radiotherapy, diabetes, obesity and increased breast volume^28–34^. These risk factors may influence the blood supply of the flap. If the perfusion of the mastectomy skin flap with sufficient oxygenated hemoglobin is compromised, necrosis may ensue. The use of vasodilators to treat vasospasm may reduce the necrosis rate of skin flaps^13,17^. As a direct vasodilator, NTG has been shown to reduce the necrosis rate of random flaps^12,17^. Papaverine is considered an effective spasmolytic agent because of its quick onset and suitable duration of action. Ricci reported that substituting topical NTG for papaverine to treat vasospasm during a shortage was not associated with an increased rate of flap loss or return to the operating room, making NTG a safe alternative to papaverine after breast reconstruction^18^.

The ultimate benefits of NTG use for mastectomy may be a decreased rate of MFN. MFN is one of the most severe complications leading to surgical failure in mastectomy. Reconstruction after mastectomy is gaining popularity, and a meta-analysis reported by Basta *et al*. stated that IBR was associated with an elevated risk of MFN and reoperation^10^. Therefore, in this study, IBR was separated from mastectomy alone for the analysis. In our study, the pooled results from 7074 patients indicated that NTG significantly decreased the MFN rate in both IBR and indirect reconstruction. MFN may present as partial-thickness (superficial) or full-thickness necrosis. The treatment of superficial flap necrosis is different from that of full-thickness skin flap necrosis. Nevertheless, many papers did not differentiate between partial-thickness MFN, which may be treated with observation in some cases, and full-thickness MFN, which requires debridement^10,35^. The current study analyzed those two groups separately, and the results showed that NTG could reduce the incidence of full-thickness flap necrosis. However, the drug had no significant effect on the rate of superficial flap necrosis.

Once MFN occurs, it is difficult to resolve. Debridement is inevitable when necrosis is severe, especially for full-thickness skin flap necrosis. Turin *et al*.^21^ divided debridement procedures according to whether they were performed in the office or the operating room; it was found that NTG could significantly reduce the total and in-office debridement rates but could not reduce the rate of debridement in the operating room. Gdalevitch *et al*. divided debridement cases into a local anesthesia group and a general anesthesia group^19^, finding that NTG could not reduce the debridement rate in both groups. Similar to Turin’s work, our study found that the debridement rate decreased significantly after the use of NTG (OR, 0.32; 95% CI, 0.21–0.51; P < 0.01), which suggested that the use of NTG reduced the probability of reoperation.

Implant loss is a serious complication after IBR. Unfortunately, NTG has not been shown to reduce the risk of implant loss following IBR. A meta-analysis by Basta concluded that the risk of implant loss may decrease as the quality of the mastectomy tissue increases^10^. Gdalevitch *et al*. and Turin *et al*. reported that there were no significant differences in implant loss between NTG and control groups^19,21^. The results of our meta-analysis are in agreement with the findings mentioned above.

NTG acts by releasing nitric oxide into vascular smooth muscle cells. This action stimulates the release of cGMP, causing relaxation of these muscle cells and consequent vasodilation^18^. This medication has been demonstrated to be effective in treating vasospasm in other diseases of the brain and heart, and its safety in humans has been well documented. However, attention must still be paid to NTG-related adverse reactions such as headache, dizziness and hypotension. Interestingly, this study demonstrated that there was no significant difference in drug-related adverse reactions between the NTG and control groups. Nevertheless, we recommend caution when NTG is used in patients with hypotension and those who take antihypertensive medications.

We also pooled the data on early complications, including 3 events: hematoma, seroma and infection. These complications have an important impact on patients’ postoperative recovery. The total rate of early complications was significantly lower in the NTG group than in the control group (OR, 0.59; 95% CI, 0.36–0.98; P < 0.05). Regrettably, there was no difference in individual complications between these two groups.

Some limitations of this meta-analysis should be addressed. First, the included studies used different NTG doses, and the dressing types also varied, mainly including transdermal paste and ointment. Second, most of the included articles were retrospective studies rather than RCTs. This limitation leaves a large gap in the current evidence, and further RCTs should be conducted. Both of these factors should be clarified in future studies.

## Conclusion

Compared to a control treatment, NTG can be beneficial to patients by reducing the rates of MFN, debridement and additional early complications in IBR or mastectomy alone. Meanwhile, NTG did not increase the incidence of drug-related side effects relative to a control. Thus, we believe that NTG could be recommended for wound healing after mastectomy, especially when IBR is performed.
